# Examining Relationships and Differences Between the Gastrocnemius and Soleus of the Affected and Healthy Lower Limb of Athletes with Medial Tibial Stress Syndrome

**DOI:** 10.3390/muscles5010014

**Published:** 2026-02-11

**Authors:** Anna Christakou, George Plakoutsis, Athanasios Gkagkalis, Eleftherios Paraskevopoulos

**Affiliations:** 1 Laboratory of Biomechanics, Department of Physiotherapy, University of Peloponnese, 23100 Sparta, Greece; 2 Laboratory of Advanced Physiotherapy, Department of Physiotherapy, University of West Attica, 12243 Athens, Greece; gplakoutsis@uniwa.gr; 3 Department of Physiotherapy, University of Peloponnese, 23100 Sparta, Greece; pritanissg@gmail.com; 4 School of Physical Education and Sports Science, National and Kapodistrian University of Athens, 17237 Dafne, Greece; elparaskev@phed.uoa.gr

**Keywords:** medial tibial stress syndrome, gastrocnemius, soleus, muscle strength, endurance, pain, athletes

## Abstract

Objectives: Medial tibial stress syndrome (MTSS) is a common overuse injury among athletes, yet limited evidence exists regarding muscle-specific deficits. This study aimed to (a) examine differences in gastrocnemius and soleus strength and endurance between the affected and healthy lower limb, and (b) investigate the relationships between gastrocnemius and soleus strength, endurance, and pain intensity in athletes with MTSS. Methods: Thirty athletes aged 18–40 years with MRI-confirmed MTSS, who had completed a standardized physiotherapy program, underwent isometric dynamometry and heel-rise endurance testing under knee-extended (gastrocnemius) and knee-flexed (soleus) conditions. Relative strength values and heel-rise repetitions were compared between limbs using paired *t*-tests. Correlations and regression analyses were performed between pain intensity, strength, and endurance of the affected limb using Pearson’s *r* and simple linear regression. Results: Significant deficits were found in both muscles of the affected limb, with reduced isometric strength and fewer heel-rise repetitions (gastrocnemius heel-rise: *t*(29) = −6.47, *p* < 0.001; gastrocnemius strength: *t*(29) = −6.27, *p* < 0.001; soleus heel-rise: *t*(29) = −5.37, *p* < 0.001; soleus strength: *t*(29) = −4.87, *p* < 0.001). Strong positive correlations were observed between muscle strength and endurance, while higher pain intensity was negatively associated with gastrocnemius performance. Moreover, pain intensity significantly predicted gastrocnemius strength (*F*(1,28) = 5.90, *p* = 0.022, *R*^2^ = 0.17) and endurance (*F*(1,28) = 7.56, *p* = 0.009, *R*^2^ = 0.22), but not soleus function. Physiotherapy duration, number of physiotherapy sessions, and self-reported pain improvement were not significant predictors to either soleus or gastrocnemius endurance, only training frequency emerging as the only independent variable. Conclusions: Athletes with MTSS exhibited notable impairments in plantarflexor function. These findings underscore the importance of targeted strength and endurance interventions for both the gastrocnemius and soleus muscles to optimize recovery, enhance load tolerance, and reduce recurrence risk. Future research should include pre- and post-rehabilitation assessments, larger sample sizes, and sport-specific cohorts.

## 1. Introduction

Medial tibial stress syndrome (MTSS), commonly referred to as “shin splints,” is one of the most prevalent overuse injuries of the lower limb among runners, athletes involved in jumping sports, and military personnel. It is typically characterized by pain along the posteromedial border of the tibia, triggered or aggravated by weight-bearing activities, and is attributed to repetitive loading and excessive strain on the tibial cortex, resulting in microtrauma and periosteal irritation [1]. MTSS is generally considered a chronic stress-related condition that progresses as mechanical loading exceeds the adaptive capacity of bone and surrounding tissues. Clinically, patients report diffuse pain over the middle or distal third of the tibia that worsens during physical activity and improves with rest [2].

Although the exact etiology of MTSS is multifactorial, several intrinsic and extrinsic risk factors have been implicated, including excessive foot pronation, training errors, inadequate footwear, and lower-limb muscle imbalances [3]. Current pathophysiological models propose that repeated bending of the tibia during impact activities leads to an imbalance between bone resorption and formation, creating a vulnerable cortex that may progress to a stress fracture if not recognized and managed appropriately [4]. Rather than a purely periosteal inflammatory process, MTSS is now viewed as a spectrum of bone stress injury in which structural fatigue and microdamage play a central role [4,5]. Imaging studies support this concept, demonstrating bone marrow and periosteal edema patterns consistent with stress reactions in symptomatic individuals [5].

Epidemiological data emphasize the clinical significance of MTSS. Its incidence has been reported to range from approximately 13–20% in runners to as high as 35% in military recruits, making it a major contributor to time-loss and reduced performance in physically active populations [6]. Several systematic reviews and meta-analyses have identified over one hundred potential risk factors, though only a limited number are consistently associated with MTSS in clinical practice [7,8]. Stronger evidence exists for higher body mass index (BMI), greater navicular drop, altered ankle plantarflexion and dorsiflexion range of motion, and increased hip rotation range—particularly external rotation [7,9]. Elevated BMI may increase tibial loading during impact, while excessive navicular drop and reduced medial longitudinal arch height may compromise the foot’s shock-absorbing capacity and alter tibial rotation, thereby increasing stress on the tibial cortex [8,9].

From a biomechanical perspective, the way the lower limb absorbs and dissipates ground reaction forces during running is crucial. The calf musculature—particularly the gastrocnemius and soleus—plays a key role in controlling tibial loading through plantarflexion torque and eccentric control during the stance phase. Adequate strength and endurance of these muscles are essential to buffer repetitive impacts and protect the tibia from excessive mechanical stress during high-demand activities. Previous studies have shown that individuals with MTSS exhibit reduced endurance of the plantarflexor muscle group, although these studies did not assess individual muscle contributions [10]. It has been proposed that athletes predisposed to MTSS may adopt compensatory recruitment strategies to generate sufficient plantarflexion force despite deficits in specific muscles, potentially masking clinically relevant dysfunctions when only global plantarflexor strength is assessed [10,11]. Accordingly, a more detailed evaluation of gastrocnemius and soleus muscle performance has been recommended to clarify their respective roles in the onset, persistence, and recurrence of MTSS [11].

Accurate diagnosis of MTSS relies primarily on clinical history and physical examination. Typical features include exercise-induced pain along the posteromedial tibial border and reproducible tenderness spanning at least 5 cm along the tibia [2]. The presence of more focal tenderness or atypical symptoms should prompt consideration of alternative or concomitant diagnoses such as tibial stress fracture or chronic exertional compartment syndrome [2]. Imaging is mainly used to support the diagnosis and to exclude other pathologies. Magnetic resonance imaging (MRI) is considered the most useful modality for detecting bone stress reactions, periosteal changes, and soft tissue abnormalities, although its sensitivity and specificity are moderate, and many MRI findings may also appear in asymptomatic athletes [5]. Computed tomography (CT) can demonstrate cortical osteopenia and micro-resorption cavities in more advanced stages of bone stress, offering additional insight into the severity of structural involvement [12].

The rehabilitation of MTSS is typically multimodal, aiming to reduce pain, promote recovery of bone and soft tissue, correct biomechanical abnormalities, and prevent recurrence. In the acute phase, relative rest or temporary cessation of high-impact activities, in combination with cryotherapy and analgesic or non-steroidal anti-inflammatory medication, is commonly recommended to control symptoms [13]. Physiotherapeutic interventions such as soft-tissue mobilization, massage, therapeutic ultrasound, or electrotherapy may also be applied, although current evidence does not clearly support the superiority of any single modality [14]. As symptoms subside, treatment progresses to a subacute and chronic phase, focusing on progressive loading, strengthening, and neuromuscular re-education. A gradual return to running, with careful modification of training variables (intensity, frequency, surface), is essential to allow for bone adaptation and to minimize the risk of recurrence [15,16].

Exercise-based rehabilitation frequently incorporates stretching and strengthening of the calf muscles, particularly the gastrocnemius and soleus, along with core, hip, and gluteal strengthening to optimize lower-limb alignment and kinetic chain control [17,18]. Proprioceptive and balance training further enhances neuromuscular control and may help reduce the incidence of recurrent overuse injuries [19]. Additionally, optimizing footwear and prescribing foot orthoses when appropriate can aid in impact absorption and control of excessive pronation in individuals with marked navicular drop or abnormal foot posture [8,20]. Despite these advances, limited evidence directly links specific impairments in gastrocnemius and soleus muscle strength and endurance to MTSS outcomes, and existing studies rarely differentiate between these two muscles when assessing plantarflexor performance.

Taken together, current literature underscores the multifactorial etiology of MTSS and highlights the importance of targeted rehabilitation strategies. However, there is a paucity of studies that specifically evaluate the post-rehabilitation strength and endurance of the gastrocnemius and soleus muscles in athletes with MTSS. Addressing this gap could refine rehabilitation protocols and contribute to more individualized, muscle-specific interventions aimed at reducing recurrence and facilitating safe return to sport. Therefore, the aim of the present study was to examine differences in strength and endurance of the gastrocnemius and soleus between the affected and unaffected limbs in athletes with MTSS. We hypothesized that the involved limb would demonstrate reduced strength and endurance in both muscles compared with the contralateral side. Additionally, we investigated the relationship between strength and endurance of the two muscles in the affected limb and their relationship with pain intensity.

## 2. Materials and Methods

The present study employed a cross-sectional design to compare the strength and endurance of the soleus and gastrocnemius muscles between the affected and unaffected limbs.

### 2.1. Participants

An a priori power analysis was conducted using G*Power version 3.1.9.7, which showed that at least 27 participants would be needed in a paired samples *t*-test with an 80% power for detecting a large effect and a significance criterion of α = 0.05. A convenience sample of 30 male and female athletes aged 18–40 years was recruited. All participants were involved in individual or team sports and had a medical diagnosis of medial tibial stress syndrome (MTSS) confirmed through magnetic resonance imaging by an orthopedic physician. Participants were recruited from a private physiotherapy clinic in Kozani.

The inclusion criteria were: 1. Diagnosis of MTSS by an orthopedic physician with an MRI examination, 2. Engagement in training at least three times per week for the past three years, 3. Presence of intense pain in the tibial region during or after sports activity, 4. Pain on palpation for at least 5 consecutive centimeters along the tibial border, 5. Symptom duration of at least three months and 6. Completion of the physiotherapy program at the study clinic under the supervision of the same physiotherapist.

Exclusion criteria encompassed: 1. Presence of any other acute or chronic musculoskeletal injury of the lower limbs, 2. History of lower limb surgery within the past 12 months, 3. Absence of normal balance ability and 4. Participation in another physiotherapy rehabilitation program for MTSS in the past.

This study was conducted in accordance with the ethical standards of the Declaration of Helsinki. Ethical approval was obtained from the Ethics Committee of the University of the Peloponnese (Approval Number: 12024, Date: 16 May 2025) prior to data collection. Written informed consent was obtained from all participants, and this was the only document containing personal identifying information. Participants were fully informed about the study procedures, which included the completion of a demographic questionnaire, isometric dynamometry testing of the ankle plantar flexors, and two muscle endurance tests targeting the gastrocnemius and soleus muscles. The demographic questionnaire included items on athletic background, years of sport experience, training frequency, and duration of physiotherapy. Additionally, pain improvement was assessed via a self-reported item included in the demographic questionnaire, scored on a 0–10 numerical rating scale, where 0 indicated “no pain improvement” and 10 indicated “complete pain improvement.” This score was recorded at the end of the final physiotherapy session.

### 2.2. Procedures

All participants met the diagnostic criteria for MTSS and provided written informed consent. Each participant was instructed about the testing sequence and informed that they could withdraw at any point. Data collection occurred immediately after the participant’s last physiotherapy session, following approval from the physiotherapy clinic.

In the present study, all participants followed the same rehabilitation framework in terms of core therapeutic components, although individual variations in treatment duration, dosage, and progression rate were permitted. The intervention was structured according to a common phased model, including:

(a) A standardized rehabilitation progression through acute → subacute → chronic phases;
(b) Inclusion of the same categories of interventions: symptom modulation, progressive loading, calf stretching and strengthening, neuromuscular training, and footwear advice.

The full physiotherapy program is detailed in the Supplementary Material. While components of the physiotherapy program were standardized, individual progression was clinician-determined based on patient response.

To ensure consistency across participants prior to post-rehabilitation assessment, a set of standardized clinical decision criteria was applied:

1. Pain-based criterion
  o Absence of sharp or progressive tibial pain during daily activities.
  o Pain during or after exercise rated ≤3/10 on the NRS.
2. Functional tolerance criterion
  o Ability to complete sport-specific training sessions (or modified running-based activity) without symptom exacerbation the following day.
3. Therapist-guided clinical judgment
  o Final readiness for assessment determined by the treating physiotherapist based on symptom stability and load tolerance, rather than session count alone.

Testing was conducted immediately after the final physiotherapy session, ensuring a consistent post-rehabilitation assessment timepoint.

#### Isometric Dynamometry

For the assessment of the gastrocnemius, participants lay in a supine position while the examiner stabilized the dynamometer against the plantar surface of the foot. Each participant performed three maximal isometric plantarflexion contractions, each lasting five seconds, with a three-second rest interval between attempts. Testing was conducted first on the right leg, followed by the left. For soleus assessment, the participant was positioned prone with the knee flexed to approximately 90°, a position that biomechanically minimizes the bi-articular gastrocnemius and favors soleus activation. This positioning has been shown to elicit predominantly soleus-driven contractions, with approximately 70% relative activation of the soleus based on electromyographic (EMG) evidence [21,22]. The Kinvent software 2.23.0 was used to automatically compute key outcome measures, including maximum force, mean force, rate of force development, time to peak force, inter-limb asymmetry, and fatigue indices [10].

Heel-Rise Endurance Tests

Participants performed single-leg heel-rise tests under two distinct conditions:

1. Knee Extended—to assess gastrocnemius endurance
2. Knee Flexed—to assess soleus endurance

For both tests, participants stood barefoot on one leg, with the contralateral limb relaxed and elevated. Participants could choose arms to be placed either on the hips or lightly in contact with a stable surface for balance. A metronome set to 60 beats per minute (bpm) provided a pacing cue, with one heel-rise repetition performed every two seconds. Testing continued until volitional fatigue or loss of proper technique (i.e., inability to maintain pace or form) [10]. An examiner recorded the number of completed repetitions. Minor support was allowed, such as resting one hand lightly on a wall or treatment table. The test was terminated if balance was lost or the participant could not continue.

Perceived exertion was assessed immediately after each test using the Borg Rating of Perceived Exertion (RPE) scale. All participants reported RPE values greater than 17, indicating near-maximal exertion levels [23]. Each test was performed once per limb, and an examiner remained nearby at all times to ensure participant safety.

The heel-rise endurance test has demonstrated excellent test–retest reliability in both healthy populations and under standardized protocols, with intraclass correlation coefficient (ICC) values up to 1.0 for maximum force and approximately 0.91 for total repetitions [24,25], supporting its use as a robust measure of plantarflexor endurance.

### 2.3. Main Outcome Measures

The following instruments were used for data collection:

1. Kinvent Dynamometer for isometric strength assessment of the gastrocnemius and soleus muscles. The accompanying Kinvent software 2.23.0 guided the testing procedure and provided quantitative output. The system demonstrates excellent reliability for strength assessments (ICC > 0.80) [26].
2. Heel-Rise Test performed with full knee extension and with knee flexion for selective assessment of the gastrocnemius and soleus, respectively [27]. Participants performed single-leg heel rises to failure or until a technical error occurred (loss of balance or incomplete range of motion). A metronome set to 60 beats per minute regulated the pace (one repetition every 2 s). Minimal fingertip support on a wall or bed was permitted [28].
3. Borg Rating of Perceived Exertion (RPE) Scale, ranging from 6 (no exertion) to 20 (maximal exertion), was used to assess perceived fatigue at the end of each endurance test [29]. The scale has demonstrated high reliability among athletes (ICC > 0.80) [30].
4. Numerical Rating Scale (NRS) [31] The NRS is a self-reported pain scale ranging from 0 (“no pain”) to 10 (“worst pain imaginable”). It is a valid and reliable instrument for measuring pain intensity [31]. Each participant was asked to report the worst pain they could recall prior to the start of the physiotherapy program.

### 2.4. Statistical Analysis

Descriptive statistics (means, standard deviations, and frequencies) were calculated for demographic characteristics and all study variables. The normality of all continuous variables and difference scores was assessed using the Shapiro–Wilk test.

All analyses were hypothesis-driven, based on established biomechanical and neuromuscular distinctions between the plantarflexor muscles except the additional regression models below using between-limb difference scores. Paired *t*-tests were conducted to compare gastrocnemius and soleus performance in both strength and endurance measures. A Bonferroni correction with alpha level adjustment was applied for multiple comparisons across the four *t*-tests (α = 0.05/4 = 0.0125).

Pearson correlation coefficients were computed to examine associations between pain intensity and the main outcome variables. A Bonferroni-adjusted alpha level of 0.01 (α = 0.05/5 = 0.01) was applied to account for multiple correlation tests and to control the family-wise Type I error rate.

Simple linear regression analyses were conducted to assess the predictive ability of pain intensity on the strength and endurance of the gastrocnemius and soleus muscles in the affected limb. Separate regression models were specified a priori for gastrocnemius and soleus based on their distinct biomechanical and neurophysiological functions. These analyses were hypothesis-driven, under the assumption that pain intensity would significantly predict gastrocnemius strength and endurance, consistent with pain-mediated neuromuscular inhibition and altered plantarflexor load distribution [32].

Additional regression models, which were exploratory analyses, were applied using between-limb difference scores (Δ = affected − healthy) in muscle endurance to investigate whether variability in rehabilitation exposure or training characteristics (e.g., training frequency, physiotherapy duration, number of physiotherapy sessions, and self-reported pain improvement) was associated with asymmetry in muscular endurance outcomes.

Statistical significance was set at α = 0.05. All analyses were conducted using SPSS for Windows, Version 29.0 (IBM Corp., Armonk, NY, USA).

## 3. Results

### Descriptive Statistics of the Sample

A total of 30 adult athletes (male and female) participated in the study, with a mean age of 25.9 ± 6.91 years, a mean height of 1.72 ± 0.15 m, and a mean body mass of 67.9 ± 10.35 kg (Table 1). All participants were long-term residents of the municipality of Kozani, regularly engaged in sports activities, and had completed a full physiotherapy rehabilitation program in the same physiotherapy clinic from the same physiotherapist. None had undergone lower-limb surgery in the previous 12 months. Additional descriptive statistics regarding pain intensity, improvement after physiotherapy, number of sessions, average training duration, and time since symptom onset are presented in Table 1.

All participants (100%) had a previous diagnosis of MTSS by an orthopedic physician following MRI confirmation, with a mean duration since diagnosis of 17.13 ± 8.1 months. The majority (90%) were not receiving medication.

They engaged in regular sports training. The most common activity was running (36.7%), followed by basketball (16.7%), football (13.3%), handball (6.7%), volleyball (6.7%), and other sports (3.3% each). Training frequency ranged from 1–2 sessions per week (10%) and 3–5 sessions per week (53.3%) to daily training (36.7%).

Most athletes (76.7%) reported no recent increase in training intensity or volume, and 73.3% experienced pain after exercise rather than during. A total of 76.7% reported residual symptoms.

Regarding return to sport, 96.7% of the participants had returned to activity (53.3% at a high level, 26.7% at a moderate level, and 16.7% at a low level). Seven participants (23.3%) reported coexisting musculoskeletal disorders.

Table 2 presents the frequency distribution of the demographic variables.

Table 3 reports the descriptive statistics for relative strength values (strength/body mass) of the gastrocnemius and soleus muscles, as well as the heel-rise test repetitions for affected and healthy limbs.

Normality was assessed using the Shapiro–Wilk test. All variables demonstrated normal distributions (*p* > 0.05), supporting the use of parametric statistical analysis. Statistically significant differences were identified between affected and healthy limbs across all strength and endurance variables using paired *t*-tests. Specifically, statistically significant differences were observed between (a) affected and unaffected gastrocnemius strength (*t* = − 6.27, *p* < 0.001), (b) affected and unaffected soleus strength (*t* = − 4.87, *p* < 0.001), (c) affected and unaffected gastrocnemius endurance (*t* = −6.47, *p* < 0.001), and (d) affected and unaffected soleus endurance (*t* = −5.37, *p* < 0.001). All the Cohens’ d with Hedges’ correction were very large (Table 4) [33]. To control for family-wise error across the four primary comparisons, a Bonferroni-adjusted significance level was applied (α = 0.0125). All primary comparisons remained statistically significant after correction.

Pearson r correlations revealed the following significant relationships after Bonferroni correction (*p* < 0.01):

(a) Strength and endurance correlations
  - Positive correlations between affected gastrocnemius endurance with affected soleus endurance (r = 0.81, *p* < 0.001)
  - Positive correlations between affected gastrocnemius endurance with affected gastrocnemius strength (r = 0.57, *p* < 0.001)
  - Positive correlations between affected gastrocnemius endurance with affected soleus strength (r = 0.58, *p* < 0.001)
  - Positive correlations between affected gastrocnemius strength with affected soleus strength (r = 0.89, *p* < 0.001)
  - Positive correlations between affected soleus endurance with affected gastrocnemius strength (r = 0.49, *p* = 0.006)
(b) Pain Muscular correlations

Negative correlation between affected gastrocnemius endurance and pain intensity (r = −0.47, *p* = 0.009)

The linear regression analyses showed in the affected limbs that:

(a) The pain intensity predicted the strength of gastrocnemius muscle (R = 0.41, *R*^2^ = 0.17, *F*(1,28) = 5.90, *p* < 0.022, β = −0.42, *t* = −2.43, *p* = 0.02, 95% CI = −0.24–0.002).
(b) The pain intensity predicted the endurance of gastrocnemius muscle (R = 0.47, *R*^2^ = 0.22, *F*(1,28) = 7.56, *p* < 0.009, β = −0.46, *t* = −2.78, *p* = 0.009, 95% CI = −3.32–0.51).

The pain intensity did not predict the strength and the endurance of the affected soleus muscle.

Lastly, to account for potential effects of participant heterogeneity, exploratory regression models were run using between-limb difference scores (Δ = affected − healthy) in muscle endurance as dependent variables.

- For soleus endurance, the multivariable model showed statistical significance: *R*^2^ = 0.323, *F*(4,25) = 2.98, *p* = 0.038
  o Training frequency was the only independent predictor (*p* = 0.009, *R*^2^ = 0.219)
  o Physiotherapy duration, number of sessions, and pain improvement were not significant.
- For gastrocnemius endurance, the model was not significant (*R*^2^ = 0.166, *p* = 0.317), and none of the individual predictors explained variance in endurance asymmetry.

These findings suggest that soleus endurance asymmetry may be influenced by ongoing training frequency, whereas gastrocnemius endurance appears less sensitive to the demographic variables examined.

## 4. Discussion

The aim of the present study was to (a) compare gastrocnemius and soleus muscle strength and endurance between the affected and unaffected limbs, and (b) investigate the relationship between muscle performance and pain intensity in athletes with MTSS. Although the strength and endurance of these muscles have been previously examined in athletes—primarily runners—no prior study has simultaneously investigated both characteristics across limbs and their potential association with pain.

The findings demonstrated that young, high-level athletes with MTSS exhibited significantly reduced strength and endurance in the gastrocnemius of the affected limb. Participants consistently performed fewer repetitions during the heel-rise test and showed lower isometric force during dynamometry. These results align with those of Madeley et al. [10], who reported reduced endurance of the plantarflexors in individuals with MTSS using the heel-rise test. While their study included a control group, the present study used a within-subject design, comparing affected and unaffected limbs, acknowledging that several participants experienced bilateral symptoms with varying severity.

Comparable findings were also reported by Mattock et al. [11], who assessed long-distance runners with MTSS using hand-held dynamometry and the heel-rise test. Although their sample was restricted to runners, and the present study included athletes from a range of sports, both studies identified significant strength and endurance deficits. Notably, the magnitude of performance reduction was smaller in the current study. Mattock et al. [11] also observed altered muscle morphology, including increased gastrocnemius thickness in symptomatic athletes—a dimension not explored in the present work.

The mechanisms underlying gastrocnemius weakness remain unclear. Akiyama et al. [34] reported increased shear modulus (i.e., stiffness) in several lower-limb muscles—including both heads of the gastrocnemius—using shear wave elastography in MTSS participants. Increased muscle stiffness may limit range of motion, disrupt optimal force–length relationships, raise energy expenditure, and predispose individuals to earlier fatigue [17,35], potentially contributing to the performance deficits observed in the affected limb.

Similarly, the soleus muscle showed significant strength and endurance impairments on the affected side. Participants performed fewer repetitions during the bent-knee heel-rise test and produced lower isometric force when assessed in knee flexion, which minimizes gastrocnemius contribution and isolates the soleus. These findings are consistent with those of Mattock et al. [11], who also reported compromised soleus function in MTSS athletes. However, unlike their protocol, the present study specifically tested plantarflexor performance in knee flexion. Reduced soleus diameter in symptomatic individuals has been interpreted as indicative of compensatory neuromuscular strategies, where soleus atrophy may lessen periosteal traction on the medial tibia. While this may reduce one source of traction, it may redistribute mechanical stress elsewhere, thereby exacerbating tibial loading [4].

Supporting this notion, Naderi et al. [36] found increased soleus activity during the heel-off to toe-off phases of gait and running in individuals with MTSS, suggesting compensatory activation patterns. Although elevated activation does not equate to increased strength, it may reflect altered motor strategies that lead to muscular overload. Akiyama et al. [34] similarly found increased stiffness in the soleus, reinforcing the hypothesis that altered mechanical properties and neuromuscular rigidity may contribute to functional impairments.

Furthermore, endurance-related adaptations in the soleus are likely driven by current training exposure rather than static clinical history, as skeletal muscle phenotype is highly use-dependent and rapidly modifiable with changes in training dose [37]. Frequent training may therefore preferentially enhance oxidative capacity, fatigue resistance, and neuromuscular efficiency in the soleus, amplifying endurance adaptations and asymmetry reduction over time [38]. In contrast, gastrocnemius endurance may show weaker associations with rehabilitation exposure metrics such as physiotherapy duration, number of sessions, or pain improvement. Given the gastrocnemius’ greater fast-twitch fiber composition and its functional role in high-force and explosive tasks, endurance adaptations in this muscle are likely more dependent on stimulus intensity, mechanical loading, and recovery characteristics than on exposure duration alone, indicating muscle-specific responses to training adaptation [39]. In summary the frequent, low-to-moderate intensity loading may favor endurance adaptations in the soleus, while gastrocnemius endurance may require different or more specific stimuli, making it appear less sensitive to training frequency or rehabilitation exposure metrics.

Significant positive correlations between muscle strength and endurance for both the gastrocnemius and soleus reinforce the physiological link between force production and fatigue resistance [40]. The strong associations between heel-rise performance on affected and unaffected limbs may reflect a generalized endurance capacity inherent in trained athletes [41]. In contrast, the observed negative associations between pain intensity and both strength and endurance underscore the inhibitory role of pain in optimal muscle activation [42]. These findings highlight the importance of effective pain management, as persistent discomfort may suppress neuromuscular output and delay recovery.

Furthermore, pain intensity was found to significantly predict both strength and endurance of the gastrocnemius, but not of the soleus. This may reflect differences in fiber-type composition and neuromechanical function—given the predominantly type I fiber profile of the soleus compared to the mixed fiber composition of the gastrocnemius [43]. The absence of significant associations between pain and soleus performance supports a muscle-specific mechanism of pain modulation, rather than generalized plantarflexor deconditioning [40]. Additionally, strength of both the gastrocnemius and soleus predicted their respective endurance performance, and vice versa, supporting a bidirectional relationship between force capacity and fatigue resistance—consistent with fundamental principles of muscle physiology [44].

These findings may hold clinical relevance, particularly in guiding the prevention of plantarflexor strength and endurance decline in athletes with persistent pain. Given the role of plantarflexor dysfunction in increasing tibial loading, such impairments may contribute to the chronic progression and recurrence of MTSS [11].

### 4.1. Clinical Implications

The present findings offer clinically relevant insights into persistent muscle deficits following rehabilitation in athletes with MTSS. Specifically, the results indicate measurable strength and endurance impairments in both major plantarflexor muscles—gastrocnemius and soleus—of the affected limb. Although the absolute between-limb difference in gastrocnemius strength was modest (0.038 N/kg), this corresponded to approximately 8% relative inter-limb strength asymmetry. Such magnitude falls within the range previously suggested as clinically meaningful in athletic populations, particularly in the context of repetitive loading and overuse injuries [45].

These results underscore the importance of incorporating targeted strength and endurance training for both the gastrocnemius and soleus muscles into rehabilitation protocols, with the aim of improving functional outcomes and minimizing the risk of recurrence. Furthermore, the observed negative associations between pain intensity and muscle performance may assist in the early identification of athletes at elevated risk of MTSS, highlighting the need for proactive monitoring and pain-informed intervention strategies.

### 4.2. Strengths, Limitations and Future Recommendations

Strengths of the present study include its focus on muscle-specific assessment following rehabilitation—an area with limited representation in the existing literature—and the inclusion of athletes with MRI-confirmed MTSS. The evaluation of two distinct plantarflexor muscles provided a more detailed and nuanced understanding of MTSS-related neuromuscular impairments.

However, several limitations must be acknowledged. The relatively small sample size limits the generalizability of the findings, and the heterogeneity of sport participation may introduce variability in biomechanical loading patterns. Although handheld dynamometry offers clinical practicality and acceptable reliability it does not match the precision of isokinetic dynamometry systems. Additionally, reliance on self-reported measures introduces the potential for recall bias. Importantly, the absence of pre-rehabilitation assessments precludes evaluation of changes over time and prevents conclusions regarding the effectiveness of the rehabilitation protocol. Thus, the present study does not assess rehabilitation efficacy but instead characterizes post-rehabilitation, muscle-specific outcomes in athletes who have completed a standardized program.

Another limitation is that subgroup analyses and dose–response evaluations based on rehabilitation duration, number of physiotherapy sessions, or return-to-sport level were not feasible. Although variability in treatment exposure existed, the sample size and uneven distribution across potential subgroups would have resulted in underpowered and statistically unreliable comparisons. Consequently, the analyses were restricted to group-level comparisons between affected and unaffected limbs.

Future studies should include larger sample sizes, stratified sport-specific subgroups, and pre- and post-rehabilitation testing to monitor muscle recovery trajectories. The use of isokinetic dynamometry may improve measurement precision for strength evaluation. In addition, future research should explore psychosocial variables—such as fear of re-injury and self-efficacy—that may influence rehabilitation adherence and return-to-sport outcomes [46]. Rehabilitation protocols are encouraged to incorporate targeted strength and endurance exercises for both the gastrocnemius and soleus muscles to optimize functional recovery and reduce recurrence risk.

## 5. Conclusions

The present study demonstrated that both gastrocnemius and soleus muscles in the affected lower limb of athletes with MTSS exhibit significantly reduced strength and endurance compared to the unaffected limb. These findings highlight the necessity of integrating targeted strengthening and endurance interventions for both muscles within rehabilitation protocols to optimize recovery, prevent recurrence, and facilitate safe and effective return to sport.

## Figures and Tables

**Table 1:** Descriptive Statistics of Demographical Characteristics.

Variable	Mean	SD	Minimum	Maximum
Age (years)	25.9	6.91	18	40
Height (cm)	172.18	10.56	154	202
Weight (kg)	67.9	10.35	50	95
Pain intensity (0–10)	4.93	1.63	2	8
Pain improvement (0–10)	5.87	1.96	2	10
Number of physiotherapy sessions	22.43	10.17	10	50
Mean training duration (min)	94.83	38.74	40	240
Time since symptom onset (months)	17.13	8.1	3	37

**Table 2:** Frequency Distribution of Demographic Characteristics.

Variable	Category	Frequency (n)	Percentage (%)
Diagnosis of MTSS	Yes	30	100
Medication use	Yes	3	10
	No	27	90
Regular training	Yes	30	100
Type of sport	Running	11	36.7
	Basketball	5	16.7
	Football	4	13.3
	Handball	2	6.7
	Volleyball	2	6.7
	Javelin	1	3.3
	Decathlon	1	3.3
	Hiking	1	3.3
	Tennis	1	3.3
	Running and Weight Training	1	3.3
	Running and Pilates	1	3.3
Training frequency	1–2 times/week	3	10
	3–5 times/week	16	53.3
	Daily	11	36.7
Recent increase in training load	Yes	7	23.3
	No	23	76.7
Pain occurrence	After exercise	22	73.3
	During exercise	8	26.7
Residual symptoms	Yes	23	76.7
Return to sport	None	1	3.3
	Low level	5	16.7
	Moderate level	8	26.7
	High level	16	53.3
Additional musculoskeletal disorders	Yes	7	23.3
	No	23	76.7

**Table 3:** Descriptive Statistics of Strength/Body Mass Ratios (N/kg) and Heel-Rise Performance.

Variable	Mean	SD	95% Confidence Interval	Minimum	Maximum
^1^ Strength for affected gastrocnemius (Newton/kg)	0.42	0.05	0.41–0.44	0.29	0.54
^2^ Strength for healthy gastrocnemius (Newton/kg)	0.46	0.06	0.44–0.49	0.33	0.67
^3^ Strength for affected soleus (Newton/kg)	0.34	0.05	0.32–0.36	0.21	0.50
^4^ Strength for healthy soleus (Newton/kg)	0.37	0.04	0.36–0.39	0.25	0.47
^5^ Endurance affected gastrocnemius	30.03	6.72	27.52–32.55	10	44
^6^ Endurance healthy gastrocnemius	32.83	6.60	30.37–35.30	18	47
^7^ Endurance affected soleus	20.63	6.08	18.36–22.91	8	33
^8^ Endurance healthy soleus	23.03	5.56	20.96–25.11	12	35

^1^ Relative force affected gastrocnemius; ^2^ Relative force healthy gastrocnemius; ^3^ Relative force affected soleus; ^4^ Relative force healthy soleus; ^5^ Heel-rise repetitions for affected gastrocnemius; ^6^ Heel-rise repetitions for healthy gastrocnemius; ^7^ Heel-rise repetitions for affected soleus; ^8^ Heel-rise repetitions for healthy soleus.

**Table 4:** Paired *t*-test Results Between Affected and Healthy Limbs in the measured variables.

Variable Pair	Mean Difference	SD	*t*-Value	Effect Size d with Hedges’ Correction	*p*
Strength for affected gastrocnemius—Strength for healthy gastrocnemius	−0.038	0.033	−6.27	1.15	<0.001
Strength for affected soleus— Strength for healthy soleus	−0.029	0.033	−4.87	0.96	<0.001
Endurance for affected gastrocnemius—Endurance for healthy gastrocnemius	−2.80	2.36	−6.47	1.12	<0.001
Endurance for affected soleus— Endurance for healthy soleus	−2.40	2.44	−5.37	0.87	<0.001

SD: Standard Deviation; Note: Negative values indicate lower values in the affected limb.

## Data Availability

Data is available after corresponding’s author permission due to privacy or ethical restrictions.

## Supplementary Materials

The following supporting information can be downloaded at: https://www.mdpi.com/article/10.3390/muscles5010014/s1, Physiotherapy Program.

## Abbreviations

The following abbreviations are used in this manuscript:

MTSS	Medial tibial stress syndrome
BMI	Body Mass Index
RelFG	Relative Force Gastrocnemius
RelFSP	Relative Force Soleus
HR	Heel-Rise Gastrocnemius
HRS	Heel-Rise Soleus
