# Supplementary material for: Examining Relationships and Differences Between the Gastrocnemius and Soleus of the Affected and Healthy Lower Limb of Athletes with Medial Tibial Stress Syndrome
Source: Muscles. 2026 Feb 11;5(1):14. doi: 10.3390/muscles5010014 (PMC12922045; doi:10.3390/muscles5010014)
Supplement: Supplementary file 1 [file muscles-05-00014-s001.zip › muscles-4034733-supplementary.pdf]

Physiotherapy Program

Ice, rest, stretching, and strengthening are common components of physiotherapeutic management for MTSS. In particular, during the acute phase, rest and temporary cessation of sports activities in combination with medication appear to improve symptoms within the first two to six weeks. The administration of painkillers such as NSAIDs and acetaminophen (e.g., ibuprofen, aspirin, naproxen) seems to alleviate pain after approximately twenty minutes of activity. Cryotherapy is also commonly utilized during the acute period. Ice may be applied directly to the affected area immediately after exercise for approximately 15–20 minutes, often using ice packs applied for 20 minutes at a time [1].

Other frequently used physiotherapeutic modalities during this phase include soft tissue mobilization, therapeutic massage, ultrasound, and electrotherapy, commonly with TENS. For example, ice cube massage has been applied to the entire leg for 10 minutes, followed by two-channel TENS for an additional 10 minutes (pulse rate = 150 Hz, pulse width = 150 μs) using two self-adhesive electrodes placed over the anteromedial lower leg compartments. The intensity was gradually increased until the participant's comfort threshold was reached. However, none of these methods have demonstrated superior effectiveness over others [2].

In the subacute and chronic phases, the primary goal of treatment is to improve training status and correct biomechanical deficits associated with the condition. Soft tissue injuries may be minimized by reducing the range, frequency, and duration of running. Rest from high-intensity exercise is considered an effective treatment strategy for overuse injuries [3]. A gradual return to full activity is crucial to prevent recurrence. Athletes should begin with low-intensity training, progressing gradually to uphill running with increased speed, intensity, and distance. Low-intensity activities should be maintained during recovery. Cross-training, such as jogging (e.g., 30 min/day at approximately 30% HRmax for two weeks), has been incorporated into rehabilitation protocols [4]. Activity typically resumes after approximately one week, once pain has subsided.

To avoid muscle fatigue, rehabilitation commonly includes stretching and strengthening exercises targeting the calf muscles. The most basic stretches performed are those targeting the gastrocnemius and soleus. Strengthening the trunk and hip musculature is also beneficial [5]. For example, during a standing calf stretch, the athlete places the metatarsal heads on a footstool while keeping the heel in contact with the ground and leaning forward to stretch the calf. The stretch is held for 10 seconds and repeated ten times. Developing strong core, hip, and gluteal muscles enhances running performance by improving biomechanics and reducing lower-limb overuse. Strength exercises may include 3 sets of 10 (8–12) repetitions of heel raises, seated toe raises, standing toe raises, and resisted eversion/inversion/plantarflexion/dorsiflexion.

Neuromuscular training, including proprioceptive balance exercises, further improves running mechanics and muscle stabilization, complementing strengthening and stretching to prevent reinjury [6]. Appropriate footwear and midsoles are important for proper shock absorption and injury prevention. Orthotic insoles may be used to correct excessive foot pronation or flat feet. Athletes should avoid running on hard or uneven surfaces and wear footwear with proper support. Manual therapy may also be appropriate for addressing mechanical dysfunctions of the spine, sacroiliac joint, and lower-limb muscle imbalances, and is often employed to prevent recurrence of injury [6].

References

1. Couture, C.J.; Karlson, K.A. Tibial stress injuries: decisive diagnosis and treatment of 'shin splints'. Physician Sportsmed **2002**, 30, 29-36. doi: 10.3810/psm.2002.06.337.
2. Bhandakkar, P.A.; Naqvi, W.; Burhani, T.S. Impact of physiotherapy rehabilitation on patients with bilateral osteoarthritis knee pain-a case report. J Evol Med Dent Sci **2020**, 9, 2316-2317.doi:10.14260/jemds/2020/502
3. Bele, A.W.; Qureshi, M.I.; Dhankar, S.; Seth, N. Impact of fall on anterior cruciate ligament of 33-year-old male. J Datta Meghe Institute Med Sciences University **2020**, 15, 132-133. doi:10.4103/jdmimsu.jdmimsu_184_19

4. Thacker, S.B.; Gilchrist, J.; Stroup, D.F.; Kimsey, C.D. The prevention of shin splints in sports: a systematic review of literature. Med Sci Sports Exerc. **2002**, 34, 32-40.doi: 10.1097/00005768-200201000-00006.
5. Ross, J. A review of lower limb overuse injuries during basic military training. Part 1: Types of overuse injuries. Mil. Med **1993**, 158, 410-415. PMID: 8361601.
6. Finch, P. Chronic shin splints: a review of the deep posterior compartment. Foot **1998**, 8, 119-124. doi:10.1016/S0958-2592(98)90043-8
